# Does the intervention of the school-based health promotion programme “Join the Healthy Boat” have equal or differential effects on weight status and health-related behaviours in children from a high or low socio-economic background? – A randomised controlled trial

**DOI:** 10.1186/s12889-024-20879-x

**Published:** 2024-12-05

**Authors:** Susanne Kobel, Olivia Wartha, Jürgen M. Steinacker, Jens Dreyhaupt

**Affiliations:** 1 Department of Internal Medicine II, Division of Sports and Rehabilitation, Ulm University Medical Centre, Parkstr. 11, Ulm, 89073 Germany; 2 Institute of Epidemiology and Medical Biometry, Ulm University, Schwabstr. 13, Ulm, 89075 Germany; 3 Institute for Rehabilitation Medicine Research, Ulm University, Ulm, Germany

**Keywords:** Children, Intervention, Health promotion, Prevention, Overweight, Social inequality

## Abstract

**Background:**

Worldwide, the prevalence of childhood overweight and obesity increases. Children with low socioeconomic status (SES) are more often affected by overweight and obesity. SES is also associated with health behaviours. In order to avoid health disparities, school-based health promotion programmes such as “Join the Healthy Boat” can help. Intervention outcomes can differ by SES; therefore, the purpose of this study was to investigate whether the intervention had equal or differential effects both on weight status and health-related behaviours in children from high and low SES.

**Methods:**

One thousand six hundred twenty children’s data (7.1 ± 0.6 years; 50.6% male) was analysed; anthropometric data was taken on-site, other health-related parameters, such as physical activity, nutrition, and screen media use, as well as SES were assessed subjectively. Logistic regression models and GEEs were calculated.

**Results:**

Comparisons by SES show that there were significant differences in children’s characteristics and health behaviours such as migration background, height, weight, BMI percentiles, weight status, family education level, household income, physical activity behaviour, screen media use, soft drink intake and breakfast skipping. After one year, there were no intervention effects for overweight status, physical activity, or screen media use, but children with high SES in the intervention group skipped breakfast significantly less often than in the control group (5.34 [1.44;19.85], *p* = 0.01). Parental education level and household income were also assessed separately, with similar results. Interaction analysis revealed no significant effects apart from an interaction effect for breakfast behaviour (*p* = 0.02).

**Conclusions:**

The health-promotion programme “Join the Healthy Boat” has the potential to promote children’s health via a school-based intervention and therefore, reach all children independent from their background. This study shows vast gaps between several health behaviours of primary school children depending on their SES, favouring those children coming from more privileged families. A longer implementation may possibly show more effects.

**Trial registration:**

DRKS00000949.

## Background

Worldwide, an increase in prevalence of childhood overweight and obesity [1–3] as well as its associated chronic diseases during adulthood has been observed [4–8]. Overweight and obesity are usually caused by multifactorial lifestyle choices, including unhealthy dietary habits (intake of sugar-sweetened beverages and skipping breakfast), low levels of physical activity, and too much time spent sedentary [9]. Obesity in children and adolescents is linked to hypertension, dyslipidemias, early atherosclerotic lesions, prediabetes and type 2 diabetes, and many other medical, psychological, and social complications [2, 10]. Further, obese children are approximately five times more likely to be obese in adulthood than their normal weight counterparts [11]. In Germany, 15.4% of children and adolescents, aged 3–17 years are overweight and 5.9% are obese [12].

Children and adolescents with low socioeconomic status (SES) are disproportionately more often affected by overweight and obesity than their peers from high-SES families [12–17]. SES is associated with children’s body mass index as well as their health behaviours [18]. For example, children from low-SES families spend more time being sedentary [18], have a significantly lower fruit and vegetables consumption [19–22], a significantly higher prevalence of low physical activity [23], and show a higher BMI than children with medium and high SES [18]. This association may, at least partially, be mediated by health literacy [24] but mostly, educational level and household income are the two major indicators of SES affecting children’s health behaviours [25]. Bambra addresses in her work numerous theories of healthy inequalities, which highlight the complexity of this phenomenon but also its importance to public health [26].

In order to avoid health disparities, it is important to offer children setting-based health promotion. School-based approaches have the advantage that those children who need it most are reachable, because the majority of children receive their primary education in public schools [27]. Furthermore, in Germany, health promotion is an integrated part of the school curriculum, which means that there are qualified teachers and an existing culture for teaching and learning activities related to health and physical activity [28].

One programme addressing regular physical activity, a healthy diet, and a reduction in screen media use in order to promote a healthy lifestyle in primary school children in southwest Germany is “Join the Healthy Boat” (for more detailed information see [29]). In order to minimise barriers in implementation and initiation of behaviour change, the programme’s contents and materials are integrated into the primary school curriculum focusing on action alternatives to promote behaviour change towards more physical activity, less time spent with screen media, and a more healthy diet, especially targeting a reduction of soft drink consumption and breakfast skipping. Teachers receive training courses and free ready-to-use teaching materials in order to deliver lessons over the course of one school year and incorporate several activity breaks per school day.

In order to find out whether the intended outcomes were achieved a large-scale evaluation was carried out. However, since outcomes of intervention studies can differ in socioeconomically challenged populations, the purpose of this study was to investigate whether the intervention had equal or differential effects both on weight status and health-related behaviours in children from high and low socio-economic background.

## Methods

### Intervention and evaluation design

The school-based, teacher-centred intervention was developed using the intervention mapping approach [30] and is based on Bandura’s social cognitive theory [31] and Bronfenbrenner’s socio-ecological approach [32]. The programme’s primary aim is to promote a healthy lifestyle in children. Key aspects of the intervention are more physical activity, less time with screen media, and a more healthy diet, especially targeting a reduction of soft drink consumption and a decrease of breakfast skipping. Within in-service training courses, teachers are provided with all necessary ready-to-use materials and background knowledge in order to implement the programme within the classroom curriculum. Materials include one lesson per week (on physical activity, diet, or screen media use) and daily exercise breaks of 10–15 min. To include parents into the intervention, parents’ nights, regular parents’ letters and so-called family homework are provided; the latter require joint efforts of parents and child to solve the given tasks. Further information on development, materials, implementation, and recruitment of teachers and pupils can be found elsewhere [29].

In order to evaluate this intervention, a prospective, stratified, cluster-randomised, and longitudinal study with intervention and control group was conducted. The intervention started in the intervention group straight after baseline measurements were taken, whereas the control group followed the regular school curriculum. Follow-up measurements were taken after twelve months. Details on teacher and pupil recruiting, teaching materials as well as organisation of randomisation and evaluation have been published elsewhere [29]. The study was approved by the University’s Ethics Committee and the Ministry of Culture and Education; it is also registered at the German Clinical Trials Register (DRKS-ID: DRKS00000949).

### Participants

One thousand nine hundred forty-three primary school children (7.1 ± 0.6 years; 51.2% male) in 154 classes (80 classes in the intervention group; 74 classes in the control group) participated in the evaluation study of the programme, which took place prior to the COVID-19 pandemic. 1736 of them were assessed one year after again. For the analyses, only data was taken into account of those children, who participated at baseline and follow-up as well as whose parents provided details on their socio-economic situation. Therefore, 1620 children’s data (7.1 ± 0.6 years; 50.6% male) was analysed for this research question. Parents provided written and informed consent and children their assent to taking part in the study prior to any data collection.

### Instruments

Anthropometric data (children’s height (cm) and body mass (kg)) was objectively assessed; trained technicians took the measurements during a school visit according to ISAK-standards [33, 34] using stadiometers and calibrated electronic scales (Seca 213 and Seca 862, respectively, Seca Weighing and Measuring Systems, Hamburg, Germany). Children’s weight status was determined using national reference data [35] after their BMI was converted to age- and gender-specific BMI percentiles. Children with a BMI above the 90th percentile were classed as being overweight; children with a BMI above the 97th percentile were regarded as obese [35].

Other health-related parameters such as daily screen media time, physical activity behaviours, soft drink consumption, and breakfast patterns as well as parental education levels and their household income were assessed using a parental questionnaire. The questions are based on those used in instruments of the German Health Interview and Examination Survey for Children and Adolescents (KiGGS), which assessed health behaviour in a large national sample [36]. Physical activity for instance was assessed using an 8-point-scale (0 to 7 days) on which parents could rate their children’s daily physical activity in a moderate to vigorous intensity. Screen media use was assessed for weekend and weekdays separately and parents could estimate how many minutes their child uses screen media per day on a 7-point-scale (0 min to more than 4 h). Total screen media use was calculated using a 5:2 ratio for weekday and weekend days. Soft drink consumption was assessed for at school and out of school with a 6-point-scale on how many times soft drinks were consumed (never to more than once per day). Breakfast behaviour was assessed using a 4-point-scale asking whether the child eats breakfast in the morning (never, rarely, often, always).

### Data analysis

Descriptive statistics were calculated (mean values and standard deviations) for continuous data. For categorical data, Fisher’s exact test as well as bivariate analyses were used for the detection of group differences between children from low and high socioeconomic backgrounds at baseline. To determine children’s socio-economic background, national guidelines were used [37]. Parental education level was taken into account (the highest of both parents or the level of the single-parent) as well as the family’s net household income. Parental education was dichotomised in primary and secondary education level vs. tertiary education level (i.e. high school education vs. no high school education). Monthly household income was assessed using seven categories (below 1250 EUR to 5000 EUR or more) and subsequently dichotomised in low income (below 1750 EUR per month) and medium and high income (1750 EUR or more). Both variables – parental education and household income – were taken into account for children’s socioeconomic status, but also analysed separately. In order to take children’s migration background into account, they were classified as having a migration background if at least one parent was born abroad, or the child was spoken to in another language than German in the first three years of life.

For further statistical analysis, physical activity was dichotomised by engagement on most days per week (i.e. four days or more) of at least 60 min of moderate to vigorous physical activity (MVPA) [38]. Time using screen media was dichotomised using a cut-off point of one hour per day based on national recommendations [39]. Parental data providing information on soft drink consumption was dichotomised by consuming soft drinks more than once versus less than once per week (median split). The frequency of having breakfast prior to going to school was also dichotomised as “often/always” versus “never/rarely”. Subsequently, logistic regression models were used to determine odds ratios (OR) for all health outcomes. In order to take a possible school effect into account Generalised Estimating Equations (GEEs) were used for all health outcomes. Further, in order to analyse whether the intervention could possibly increase or decrease social inequalities, GEE models with interaction were calculated. Interaction was defined as outcome*time.

Statistics were performed using SPSS Statistics 25 (SPSS Inc., Chicago, IL, US) and SAS, version 9.4 (SAS Institute, Cary, NC, US) with a two-sided significance level set to α = 0.05 and 95% confidence intervals were calculated.

## Results

A summary of participant’s baseline characteristics is shown in Table 1. No significant gender differences were found for height, weight, and BMI percentiles. The prevalence of overweight including obesity is 8.8% and of obesity alone 3.4%. Group comparing to check if the randomisation was successful revealed no differences between control and intervention group for all relevant variables with the exception of migration background, which was significantly higher in the intervention group (*p* < 0.01). At baseline, there were significant differences in children’s characteristics and health behaviours in nearly all analysed variables, when comparing children with a high socioeconomic status with those with a low socioeconomic status (see Table 1).

**Table 1:** Baseline characteristics of participants in the “Join the Healthy Boat” study

	Missing Values	Total (*n* = 1620)		High SES (*n* = 445^a^)		Low SES (*n* = 987^a^)		Difference btw. high and low SES
	n	Intervention (*n* = 895)	Control (*n* = 725)	Intervention (*n* = 246)	Control (*n* = 199)	Intervention (*n* = 533)	Control (*n* = 454)	*p*
Age, years [m (sd)]		7.1 (0.6)	7.1 (0.6)	7.0 (0.6)	7.0 (0.6)	7.1 (0.6)	7.1 (0.6)	< 0.001
Boys [n (%)]		443 (49.5)	376 (51.9)	123 (50.0)	104 (52.3)	263 (49.3)	239 (52.6)	0.528
Migration background [n (%)]	55	297 (34.4)	190 (27.1)	53 (21.8)	39 (19.8)	205 (40.3)	140 (32.3)	< 0.001
**Anthropometry**								
Height, cm [m (sd)]	44	123.9 (6.4)	123.8 (6.1)	123.5 (6.2)	123.5 (5.8)	124.3 (6.5)	124.0 (6.3)	0.036
Weight, kg [m (sd)]	45	24.7 (5.1)	24.4 (4.4)	23.7 (3.8)	23.8 (3.8)	25.3 (5.7)	24.6 (4.5)	0.003
BMIPERC [m (sd)]	45	48.7 (27.8)	47.6 (26.7)	43.3 (24.7)	44.1 (26.6)	51.6 (28.6)	48.6 (26.6)	< 0.001
Overweight and obesity [n (%)]	45	85 (9.8)	53 (7.5)	8 (3.4)	13 (6.6)	69 (13.3)	35 (7.9)	0.008
**Parental characteristics**								
Tertiary family educational level [n (%)]		293 (32.7)	229 (31.6)	246 (100.0)	199 (100.0)	14 (2.6)	12 (2.6)	< 0.001
Low household income [n (%)]	188	101 (13.0)	83 (12.7)	0 (0.0)	0 (0.0)	101 (18.9)	83 (18.3)	< 0.001
**Health and lifestyle characteristics**								
MVPA on ≥ 4 days/week ≥ 60 min/day [n (%)]	76	226 (26.5)	189 (27.4)	82 (34.0)	60 (30.6)	122 (24.1)	112 (25.9)	0.011
Screen media use > 1 h/day [n (%)]	13	132 (14.8)	92 (12.8)	15 (6.1)	16 (8.1)	98 (18.5)	62 (13.7)	0.020
Soft drink intake > 1 time/week [n (%)]	8	222 (24.9)	169 (23.5)	27 (11.0)	25 (12.6)	162 (30.5)	124 (27.5)	0.571
Breakfast skipping [n (%)]	6	112 (12.5)	87 (12.1)	13 (5.3)	10 (5.1)	85 (16.0)	70 (15.5)	< 0.001

*m (sd)* mean (standard deviation), *SES* Socio-economic background (as a combination of parental education level and household income), *BMIPERC* BMI percentiles, *MVPA* Moderate to vigorous physical activity

^a^One hundred eighty-eight children could not be allocated to either high or low SES as data on household income was missing. They still remain in the “total” group since independent analyses of both variables used to define SES (parental education and household income) were performed, too

Table 2 shows participant’s characteristics after one year, at follow-up, separated into control and intervention group as well as into children from a low socio-economic background and a high socio-economic background.

**Table 2:** Follow-up characteristics of participants in the “Join the Healthy Boat” study

	Missing Values	Total (*n* = 1540)		High SES (*n* = 426^a^)		Low SES (*n* = 938^a^)		Difference btw. high and low SES
	n	Intervention (*n* = 850)	Control (*n* = 690)	Intervention (*n* = 237)	Control (*n* = 189)	Intervention (*n* = 506)	Control (*n* = 432)	*p*
Age, years [m (sd)]		8.1 (0.6)	8.1 (0.6)	8.0 (0.6)	8.0 (0.6)	8.1 (0.7)	8.11 (0.7)	0.027
**Anthropometry**								
Height, cm [m (sd)]	28	129.9 (6.6)	130.0 (6.4)	129.7 (6.4)	129.5 (6.3)	130.2 (6.8)	130.2 (6.5)	0.098
Weight, kg [m (sd)]	33	28.0 (6.6)	27.6 (5.3)	27.0 (6.4)	26.9 (4.7)	28.6 (6.8)	27.9 (5.5)	< 0.001
BMIPERC [m (sd)]	33	48.9 (28.8)	47.2 (27.7)	43.9 (26.7)	43.8 (27.6)	51.5 (29.4)	48.5 (27.7)	< 0.001
Overweight and obesity [n (%)]	33	93 (11.2)	60 (8.9)	12 (5.1)	14 (7.6)	72 (14.5)	40 (9.4)	< 0.001
**Health and lifestyle characteristics**								
MVPA on ≥ 4 days/week ≥ 60 min/day [n (%)]	158	217 (28.7)	169 (27.0)	82 (36.6)	60 (34.3)	111 (25.3)	94 (24.0)	< 0.001
Screen media use > 1 h/day [n (%)]	121	92 (11.9)	93 (14.4)	16 (7.0)	16 (8.9)	64 (14.2)	62 (15.5)	< 0.001
Soft drink intake > 1 time/week [n (%)]	124	159 (20.6)	138 (21.4)	31 (13.6)	19 (10.5)	107 (23.9)	101 (25.2)	< 0.001
Breakfast skipping [n (%)]	123	86 (11.2)	84 (13.0)	6 (2.6)	14 (7.8)	69 (15.4)	63 (15.7)	< 0.001

*m (sd)* mean (standard deviation), *SES* Socio-economic background (as a combination of parental education level and household income), *BMIPERC* BMI percentiles, *MVPA* Moderate to vigorous physical activity

^a^One hundred seventy-six children could not be allocated to either high or low SES as data on household income was missing. They still remain in the “total” group since independent analyses of both variables used to define SES (parental education and household income) were performed, too

### Weight status

At baseline, there were significant differences in BMI percentiles and weight status when comparing children with low and high SES (baseline BMI percentiles: mean = 50.2 (SD 27.7) and mean = 46.3 (SD 25.5) for low and high SES, respectively (t 2.687, *p* < 0.001) and baseline weight status (overweight): *n* = 104 (10.8%) and *n* = 21 (4.8%) for low and high SES, respectively (t 1.450, *p* < 0.001). Further, there was a significant difference in weight status in control and intervention group in those children with a low SES, with significantly more children being overweight in the intervention group (7.9% vs. 13.3% for control and intervention group, respectively; *p* < 0.05).

After one year, there was no difference in BMI percentiles or weight status in those children with a high SES when comparing control and intervention group. For children with a low SES, there remained a significant difference in weight status at follow-up (*p* < 0.001) when comparing control and intervention group. However, whereas the percentage of children with a low SES who are overweight and/or obese stayed virtually the same in the intervention group (13.3% and 14.5% at baseline and follow-up, respectively), it increased significantly in the control group (7.9 and 9.4%; *p* < 0.001). Logistic regression models showed that, if controlling for baseline data, the significant group difference persisted for weight status in children with low SES (OR 1.63 [1.08; 2.45], *p* = 0.02). This is especially visible for boys with a low SES, as well as second graders (boys and girls) with a low SES (*p* < 0.05).

In order to check for a possible school effect and to control for age, gender, and migration background, GEE models were calculated where the intervention effect for children with low SES lost its significance (see Table 3).

**Table 3:** Intervention effects at follow-up in children with a low SES, calculated using GEEs

	*n*^*a*^	*OR*^*b*^	*p*	*95% CI*
**Weight status**				
Overweight and obesity	862	1.43	0.31	0.72; 2.84
**Physical activity**				
MVPA on ≥ 4 days/week ≥ 60 min	762	1.12	0.53	0.78; 1.60
**Screen media use**				
Screen media ≥ 1h/day	810	0.75	0.16	0.51; 1.12
**Soft drink intake**				
Soft drinks ≥ 1 time/week	810	0.90	0.61	0.61; 1.33
**Breakfast habits**				
Skipping breakfast	812	0.87	0.64	0.48; 1.57

*OR* Odds ratio, *CI* Confidence interval, *MVPA* Moderate to vigorous physical activity

^a^only cases with baseline and follow-up data

^b^adjusted for baseline values, age, gender, and migration background; school was treated as cluster in each GEE model

### Physical activity

Although there was a significant difference when comparing children with low and high SES with regards to their physical activity behaviour at baseline and follow-up (baseline: *n* = 234 (24.9%) and *n* = 142 (32.5%) for low and high SES, respectively; *p* = 0.011 and *p* < 0.001, for baseline and follow-up, respectively; s. Tables 1 and 2), after one year, there were also no intervention effects for children’s physical activity, neither for children with low nor with high SES (s. Tables 3 and 4).

**Table 4:** Intervention effects at follow-up in children with a high SES, calculated using GGEs

	*n*^*a*^	*OR*^*b*^	*p*	*95% CI*
**Weight status**				
Overweight and obesity	403	1.32	0.70	0.32; 5.35
**Physical activity**				
MVPA on ≥ 4 days/week ≥ 60 min	388	1.14	0.60	0.71; 1.83
**Screen media use**				
Screen media ≥ 1h/day	401	0.84	0.76	0.28; 2.53
**Soft drink intake**				
Soft drinks ≥ 1 time/week	403	1.40	0.38	0.67; 2.93
**Breakfast habits**				
Skipping breakfast	402	5.34	**0.01**	1.44; 19.85

*OR* Odds ratio, *CI* Confidence interval, *MVPA* Moderate to vigorous physical activity

^a^only cases with baseline and follow-up data

^b^adjusted for baseline values, age, gender, and migration background; school was treated as cluster in each GEE model

### Screen media use

At baseline as well as at follow-up, twice as many children with a low SES used screen media for more than one hour per day, than children with a high SES (baseline: *n* = 160 (16.3%) and *n* = 31 (7.0%), for low and high SES, respectively; *p* = 0.020 and *p* < 0.001, for baseline and follow-up, respectively). However, after one year, there were also no intervention effects for children’s screen media use, neither for children with low nor with high SES (s. Tables 3 and 4).

### Nutrition

There was a significant difference in breakfast skipping at baseline, when comparing children with low and high SES (*n* = 155 (15.8%) and *n* = 23 (5.2%), respectively; *p* < 0.001; s. Table 1) but no difference between control and intervention group in either children with low or high SES. At follow-up, this difference remained equally high (4.9% of children with high SES went to school without breakfast, whereas 15.5% of children with low SES regularly skipped breakfast, *p* < 0.001). Whilst there was no intervention effect observed in children with low SES, there was a significant intervention effect in children with high SES (s. Table 4), with significantly less children skipping breakfast in the intervention group, compared to control group (2.6% vs. 7.8% for intervention and control group, respectively; *p* < 0.01), even if controlling for baseline values, age, gender, and migration background.

Children with a low SES showed a little less breakfast skipping in the intervention group at follow-up, compared to baseline and control group, but no statistical significance could be reached.

Another nutritional aspect analysed was children’s soft drink consumption. Although there was a significant difference when comparing children with low and high SES with regards to their soft drink intake at follow-up (s. Table 2; *p* < 0.001), there were also no intervention effects for children’s soft drink intake, neither for children with low nor with high SES (s. Tables 3 and 4).

### Detailed analyses of parental education level and household income

Since parental educational level and household income are the two major indicators of SES affecting children’s health behaviours [25], both were analysed separately.

### Parental education level and household income

Comparing tertiary parental education level with primary and secondary parental education level, showed significant intervention effects screen media use in girls from families with lower parental education level (OR 0.52 [0.29; 0.94], *p* = 0.031). For boys from a family with tertiary education level, significant intervention effects were shown for breakfast skipping (OR 5.25 [1.19; 23.15], *p* = 0.028).

Comparing low household income (below 1750 EUR / month) with medium and high household income, showed significant intervention effects for children’s physical activity levels (OR 3.74 [1.40; 9.98], *p* = 0.009) if they came from a family with low household income. There was also a tendency towards less breakfast skipping (only for first graders: OR 0.11 [0.01; 1.17], *p* = 0.067).

### Interaction analysis

In order to analyse whether the intervention could possibly increase or decrease social inequalities, GEE models with interaction were calculated. Significant interaction effects could only be found for breakfast behaviour (*p* = 0.023). Here, the changes from baseline to follow-up are of a different magnitude (low SES: control vs. intervention: -0.18% vs. 0.58%; high SES: control vs. intervention: -2.73% vs. 2.41%).

## Discussion

In order to find out whether the health-promotion programme “Join the Healthy Boat” achieved the intended health outcomes in children with low and high SES, this cluster-randomised study was carried out. At baseline, as well as at follow-up, there are significant differences between children from low-SES families compared to high-SES families in all assessed health behaviours as well as their body composition. After a one-year intervention, children with a high SES background showed a significant intervention effect for breakfast skipping. It is known that health is affected by a wide range of social and economic determinants, leading to inequalities in disease experience, and life expectancy [40, 41]. Children and adolescents from low-SES families are significantly more often affected by overweight and obesity and show more unhealthy behaviours than their peers from high-SES families [12–17]. Longer intervention periods may be more beneficial [43], however, only few studies report effects for SES subgroups. According to a large review of interventions preventing childhood obesity [43], only 7 to 12% of studies (depending on analysed age group) analysed intervention effects for children with high and low SES separately. Results are very heterogeneous; some interventions showed greater and more favourable effects in children from families with high SES [44–47], one study reported reduced zBMI in low-SES children [48], others reported no interaction between SES and BMI [42, 49]. It is known that children from a low socio-economic background are considerably more often affected by obesity; in a national, representative sample including 1799 girls and 1762 boys, aged between three and 17 years, children with low SES were four times as often affected by obesity as children from families with high SES [12]. Against this background, health promotion and prevention measures that contribute to the reduction of overweight and obesity prevalences as well as a health inequality in more deprived children must continue. Although SES is a multi-faceted concept, there is no standardised operationalisation of SES. Commonly, it is distinguished between individuals with a “high” or “low” socioeconomic background depending on their household income and / or highest level of education attained [50]. Therefore, in addition to comparing children from high-SES families with children from low-SES families, both aspects (parental education level and household income) were also analysed individually.

Considering parental education level only, children from families with low education level, showed significant intervention effects for screen media use if considering girls only. Whereas there was no difference between intervention and control group when comparing girls from a high educational background (8.2% of girls used screen media for more than one hour per day in control and intervention group), there was a significant difference when comparing girls from a family with low education level. In the control group, 17.4% of girls used screen media for more than one hour per day whereas in the intervention group 13.8% of girls used screen media for the same amount of time per day. Although approximately twice as many children from a low SES background (and also from families who are not classed as having a tertiary education level) use more screen media per day than recommended [39], which has been reported previously [51], it seems that the intervention of “Join the Healthy Boat” addressed screen media use reduction in a way that affected girls from low-SES families. Throughout the intervention year, screen media reduction is mainly thematised using action alternatives, i.e. children were introduced to new and other ideas of what to do instead of using screen media. Most ideas, children get encouraged to try out are designed to spend their leisure time more actively [52], which obviously also inspired girls from families with a high education level. Looking at families with tertiary education level, boys seemed to benefit most from intervention elements addressing breakfast skipping.

Further, children growing up in a household with low income, displayed significantly more physical activity if in the intervention group (31.9% met the WHO guidelines [38] on most days of the week) compared to the control group that followed the regular curriculum (13.6% met the WHO guidelines [38] on most days of the week).

It has been implied that parental educational level is a stronger predictor of children’s health behaviours than family household income [53]. However, it has been shown that both, parental education and household income, are strongly related to children’s risk of obesity, with children of parents with low or medium education being 55.3% more likely to be overweight or obese and children of families with a low household income having 96.2% higher odds of being overweight [54]. Similarly, in this study, children with low SES showed significantly more often unfavourable health behaviours, such as less physical activity (one-third vs. one quarter of children meeting the WHO guidelines [38]), approximately twice as much screen media use as well as a significantly higher intake of sugar-sweetened beverages and leaving for school with not having had breakfast. This goes in line with results of a large national survey, which reported that children with a low SES are more likely to have an unhealthy diet, are less likely to engage in sufficient physical activity, and are more likely to be overweight or obese than their peers from high-SES families [55]. A recent meta-analysis investigating the effect of breakfast on childhood obesity showed that children who skipped breakfast had a significantly higher prevalence of overweight and obesity than those who ate breakfast regularly [56]. Further has been shown that children who live in areas with poor economic conditions are more likely to skip breakfast, thus leading to overweight and obesity [57]. Whereas a study across 12 countries showed that amongst others socioeconomic factors and the accessibility of school breakfast programmes affect the frequency of children eating breakfast [58].

Therefore, and in order to not let this social and health inequity grow, interventions that start early are vital. Especially if setting- or school-based, where the majority of children can be reached, independent of their background, such interventions can give children the chance for a healthy upbringing.

It was also looked at whether the intervention of “Join the Healthy Boat” could increase or decrease social inequalities. Although significant interaction effects could only be found for breakfast behaviour, which is most likely due to the different intervention effects in the subgroups of children with high and low SES. In the subgroup with children from a high socio-economic background, the changes from baseline to follow-up are of a different magnitude, which is presumably the reason why the interaction term became significant. Although, it is uncertain whether the effect of the intervention is affected by social background, rather than the intervention affecting social inequalities. Therefore, there is no evidence that the intervention could reduce social inequalities in weight status, physical activity, screen time and diet (apart from breakfast skipping).

However, according to a microsimulation model, health gains from interventions targeting children’s health behaviours occur in the long term [59], yet this intervention only lasted one school year. “Join the Healthy Boat” as a programme on the other hand, covers the entire period of primary school (four years). Still, investigation was only possible during one year due to the waiting control group starting the intervention the following year. In addition, the fact that follow-up measurements were carried out after a six-week school break (summer holidays) may have led to a dilution of the results. Then again, the study’s large sample size probably increased the likelihood of having sufficient power to detect intervention effects, yet, some sub-groups were rather small, which is also visible in the width of some confidence intervals. Therefore, those results can only be interpreted cautiously. Additionally, the use of subjective measures to assess physical activity, screen media use, and drinking/eating behaviour and the associated recall biases is a limitation of this study. Further, voluntary participation in the study may have led to an increased social desirability bias as awareness was raised for the importance of health promotion. Even though a major strength of this study is its randomised controlled design, the teachers in the control group were also very health conscious and will not have been “inactive”, which might have led to a strong contamination with other efforts to promote children’s health in the control group.

## Conclusions

The health-promotion programme “Join the Healthy Boat” has the potential to promote children’s health via a school-based, teacher-centred intervention and therefore may reach children independent from their background. This study shows that there are vast gaps between several health behaviours of primary school children depending on their SES, favouring those children coming from more privileged families. Nevertheless, even after one school year, the intervention showed different effects on breakfast habits depending on children’s socio-economic background, as well as screen media use and physical activity engagement, when considering subgroups. Therefore, if implemented for a longer period, “Join the Healthy Boat” may offer the possibility to not let social and health inequity grow but reach children from all backgrounds.

## Data Availability

The datasets used and analysed during the current study are available from the corresponding author on reasonable request.

## Abbreviations

SES: Socioeconomic status
GEE: Generalised Estimating Equations
cm: Centimetre
kg: Kilogram
BMI: Body Mass Index
BMIPERC: BMI percentiles
KiGGS: German Health Interview and Examination Survey for Children and Adolescents
MVPA: Moderate to vigorous physical activity
OR: Odds ratios
CI: Confidence interval
n: Number
m: Mean
sd: Standard deviation
EUR: Euro (currency)
